# First direct evidence of adult European eels migrating to their breeding place in the Sargasso Sea

**DOI:** 10.1038/s41598-022-19248-8

**Published:** 2022-10-13

**Authors:** Rosalind M. Wright, Adam T. Piper, Kim Aarestrup, Jose M. N. Azevedo, George Cowan, Andy Don, Matthew Gollock, Sara Rodriguez Ramallo, Randolph Velterop, Alan Walker, Håkan Westerberg, David Righton

**Affiliations:** 1 Environment Agency, Threshelfords Business Park, Feering, Essex, CO5 9SE UK; 2 Institute of Zoology, Regents Park, London, NW1 4RY UK; 3 Technical University of Denmark, National Institute of Aquatic Resources, Vejlsoevej 39, 8600 Silkeborg, Denmark; 4 Centre for Ecology, Evolution and Environmental Changes and Faculdade de Ciências E Tecnologia, Universidade Dos Açores, Rua Mãe de Deus, 9500-321 Ponta Delgada, Azores Portugal; 5 Raufarhöfn, Iceland; 6 Environment Agency, Rivers House, East Quay, Bridgwater, TA6 4YS UK; 7 Zoological Society of London, Regents Park, London, NW1 4RY UK; 8 Natural England, Sterling House, Dix’s Field, Exeter, EX1 1QA UK; 9 The Centre for Environment, Fisheries and Aquaculture Science, Pakefield Road, Lowestoft, Suffolk, NR33 0HT UK; 10 Department of Aquatic Resources, Institute of Freshwater Research, Swedish University of Agricultural Sciences, Stångholmsvägen 2, 178 93 Drottningholm, Sweden

**Keywords:** Ecology, Ecology, Environmental sciences, Ocean sciences

## Abstract

The European eel (*Anguilla anguilla*) is critically endangered (according to the most recent IUCN assessment) and has suffered a 95% decline in recruitment since the 1980s, attributed in part to factors occurring during the marine phases of its life-cycle. As an adult, the European eel undertakes the longest spawning migration of all anguillid eels, a distance of 5000 to 10,000 km across the Atlantic Ocean to the Sargasso Sea. However, despite the passage of almost 100 years since Johannes Schmidt proposed the Sargasso Sea as the breeding place of European eels on the basis of larval surveys, no eggs or spawning adults have ever been sampled there to confirm this. Fundamental questions therefore remain about the oceanic migration of adult eels, including navigation mechanisms, the routes taken, timings of arrival, swimming speed and spawning locations. We attached satellite tags to 26 eels from rivers in the Azores archipelago and tracked them for periods between 40 and 366 days at speeds between 3 and 12 km day^−1^, and provide the first direct evidence of adult European eels reaching their presumed breeding place in the Sargasso Sea.

## Introduction

The extensive surveys of Johannes Schmidt throughout the Atlantic Ocean and Mediterranean Sea in the early twentieth century demonstrated a widespread distribution of eel larvae [leptocephali], with a concentration of the smallest specimens in the Sargasso Sea, far from their freshwater, estuarine and coastal growth habitats located in Europe and North Africa^1^. This discovery remains the single biggest step to solving the mystery of how the eels reproduce and the location of their breeding place, which has perplexed generations from Aristotle to Freud^2^. Schmidt concluded that ‘the spawning grounds comprise a restricted area in the western Atlantic, north-east and north of the West Indies, between 65° and 48° long, for here- *and here only*- are the youngest, newly hatched larvae found^1^. Since this time, other surveys have located leptocephali < 12 mm in length (< 20 days old) across a 2000 km wide region of the Sargasso Sea from 70 °W to 50 °W, but over a restricted latitude near temperature fronts of 22 to 24 °C within the subtropical convergence zone^3–5^. Most recently, even younger leptocephali (< 7 mm) have been found south of the northerly frontal zone, predominantly within the area bounded by the coordinates 31°N, 50°W and 24°N, 70°W^6^. However, even 100 years on from Schmidt’s original discovery, the inferences that can be drawn from larval data are limited, and surveys have failed to locate either adult eels or eggs. As a result, the timing and spatial extent of spawning are difficult to determine exactly^7^. The fact that very small larvae have been found in inter-annual surveys across a wide longitude range could be an indication that spawning is spatially and/or temporally variable between years^8^. Increasing knowledge on the spawning migration and the location of adult eels during the spawning period, as well as identifying the depth and oceanographic conditions for spawning, is therefore an important next step in eel research^9–13^.

**Figure 1:**
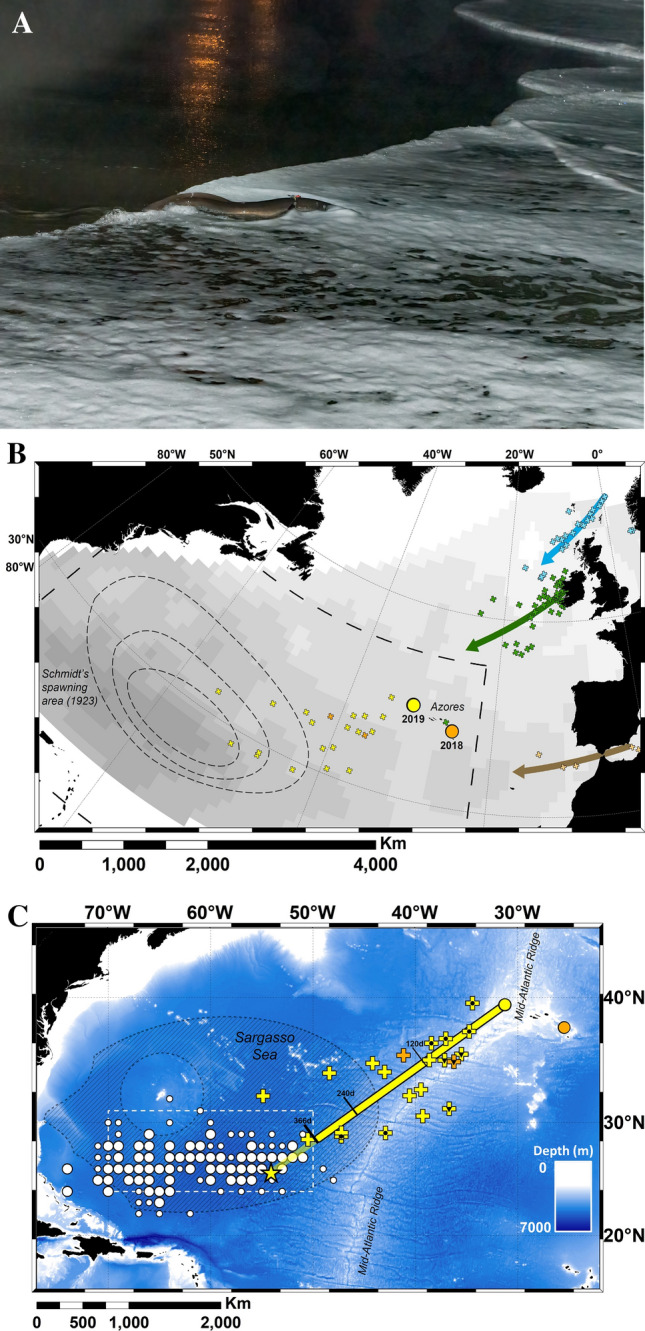
Migration of European eels from the Azores to the Sargasso Sea spawning area. (**A**) European eel fitted with a pop-up satellite tag; (**B**) historic data, showing the location of the proposed spawning area^1^ in relation to previous pop-up satellite tag positions (crosses) from eels released from three different locations in Europe^20^. Shading shows kriged minimum leptocephalus size data from ICES in a 1° grid; (**C**) pop-up data from European eels tagged at two Azores locations (circles) in November of 2018 (gold) or 2019 (yellow). Symbols within the crosses show if the tags detached from the eels prematurely, either due to exceeding the depth failsafe of 1400 m (downward triangle), or for other (unknown) reasons (circle). Predations are not shown. The average bearing of eels released in 2019 is shown as a yellow line, with intervals along the line marking the distance travelled at the average speed shown by eels at liberty for 120 day or less, at liberty for between 120 and 240 day, and at liberty for > 240 day. The yellow star shows the extrapolated position along the average bearing for a migratory period ending at the second peak spawning event after release (i.e. 14th February^20,22^, 466 day after release). White circles (at 1° grid) show where the smallest larval eels have been recorded in larval surveys conducted over the last century (large circles = larvae < 8 mm, small circles = larvae 8 mm to 12 mm), while the white dotted line shows the boundary extent of the spawning area in recent times, as presented in Miller et al*.*^6^. The hatched area shows the area defined as the Sargasso Sea Area of Collaboration^23^. The bathymetry used in the (**B**) and (**C**) is the GEBCO grid (GEBCO Compilation Group, 2020)^24^, and both figures were drawn in ESRI ArcMap 10.5, available to download at the ESRI website (https://support.esri.com/en/Products/Desktop/arcgis-desktop/arcmap/10-8-2#downloads).

Of all the anguillid species of eel, the European eel has the longest and most complex ocean migration^14,15^. Attempts have been made since the 1970s to track the oceanic migrations of eels^16^, but significant progress has only been made in the last 10 to 15 years with the advent of pop-up satellite transmitting tags (PSAT)^17^. In recent studies, pop-up tags (Fig. 1A) were used to track the migrations of more than 80 eels released from five regions within Europe: the Baltic Sea, the North Sea, the Celtic Sea, the Bay of Biscay and the western Mediterranean^18–21^. The data from the tags were used to identify migratory routes that extended up to 5000 km from release, and which suggested routes taken by eels migrating from different countries converge when passing the Azores (Fig. 1B). However, although eels were tracked for six months or more, their migration speed was insufficient to reach the Sargasso Sea for the first presumed spawning period after migration commenced, prompting the hypothesis that the spawning migration period of eels may extend to more than 18 months^20^.

To gather direct evidence on the final stage of the migration of European eels to the Sargasso Sea, (based on the assumption that European eels that spend their growth phase in the Azores would migrate to the Sargasso Sea along the same route as eels from continental Europe and North Africa), we carried out exploratory surveys for European eel in waterbodies on several Azorean islands in 2017, 2018 and 2019. Twenty-six female silver eels (mean length 911.9 mm +/- 51.8 s.d.) were captured, tagged with PSATs and released from shore in November and December 2018 and 2019 (Table 1).

**Table 1:** Metrics, distance, time and speed of PSAT tagged eels. Data from eels that were predated are not shown. If the surfacing position of tags needed to be back-calculated from surface drift, it is shown in bold against the ‘pop-up reason’.

Tag ID	Release date	Pop-up date	Pop-up reason	Length (mm)	Mass (g)	Ocular Index	No. days at liberty	Bearing (degrees)	Distance tracked (km)	Mean speed (km day^-1^)
56437	06/11/19	06/06/20	Programmed	896	1535	7.7	213	241	1198	5.6
56441	06/11/19	06/06/20	Programmed	896	1430	9.1	213	226	1160	5.4
56446	06/11/19	06/06/20	Programmed	956	1670	7.7	213	249	1638	7.7
56448	06/11/19	06/06/20	Programmed	919	1500	11.0	213	234	810	3.8
56449	06/11/19	13/05/20	**Premature**	988	1595	6.1	189	223	1572	8.3
56451	06/11/19	11/05/20	**Premature**	904	1405	7.6	187	231	1886	10.1
56452	06/11/19	06/04/20	Too deep	866	1510	8.0	152	208	1046	6.9
56453	06/11/19	06/06/20	**Programmed**	894	1635	8.3	213	232	1863	8.7
56457	06/11/19	16/02/20	Too deep	904	1605	7.2	102	227	709	7.0
56464	09/12/18	11/04/19	Too deep	990	1850	10.4	123	253	1010	8.2
56474	09/12/18	26/07/19	**Premature**	865	1385	10.3	229	260	1410	6.2
56475	06/11/19	19/01/20	**Premature**	927	1600	8.5	74	240	583	7.9
56477	06/11/19	08/12/19	**Premature**	1019	2160	7.7	32	233	380	11.9
56481	27/11/19	27/07/20	Programmed	820	1440	10.4	243	246	1252	5.2
56482	06/11/19	06/07/20	Programmed	902	2045	7.2	243	216	1230	5.1
56488	06/11/19	06/07/20	Programmed	887	1535	7.3	243	236	2148	8.8
56492	06/11/19	17/12/19	**Premature**	889	1505	7.1	41	273	272	6.6
196733	27/11/19	12/06/20	**Premature**	855	1485	12.5	198	242	711	3.6
196736	27/11/19	22/02/20	Too deep	907	1410	12.0	87	221	568	6.5
196737	27/11/19	27/11/20	Programmed	865	1345	10.9	366	249	2275	6.2
196738	27/11/19	27/11/20	Programmed	1000	1930	9.3	366	225	1048	2.9
			Mean	911.9	1599	8.9	188	236	1179	6.8
			S.D	51.8	221	1.8	89	15	564	2.2
			Max	1019	2160	12.5	366		2275	11.9

## Results

Twenty-three tags communicated via the ARGOS system (www.argos-system.org), of which 21 recorded substantive data before the tags detached 40 to 366 days after deployment (mean duration 187 day ±  89 s.d.). Two tags were assessed to have become detached from eels within a week of release either through attachment failure or predation. The tag pop-up locations confirm the south-westerly trajectory of their migration in the direction of the Sargasso Sea (bearings ranged from 208° to 273°), covering straight-line distances of 272 to 2275 km (mean 1179 km ±  564 s.d.) (Fig. 1C, Table 1). Five of the pop-up locations were within the Sargasso Sea boundaries^25,23^ and one eel was located within the presumed breeding area located between 31 °N, 50 °W and 24 °N, 70 °W^6^. Average migration speed ranged between 2.9 and 11.9 km day^−1^ (mean 6.8 km day^−1^ ±  2.2 s.d.).

## Discussion

Despite the journey for European eels from the Azores being the ‘shortest’ migration route to the Sargasso Sea at approximately 2500 km to the eastern edge of the presumed spawning area, it is still longer than the full migration route of some tropical anguillid species^14^. However, the period of time that eels leaving from the Azores have to reach the spawning area before the spawning period starts is not long: larval surveys have shown that spawning each year begins in December, peaks in February/March and extends into May^20,26^. This provides a period of between 1 and 6 months in which eels migrating from the Azores have to complete their journey and sexual maturation process and to join other spawners. However, none of the eels tagged in this study migrated fast enough (greater than 12 km day^−1^) to arrive in the spawning area before the end of the recognised spawning period. Instead, their speed over ground averaged 6.5 km day^−1^ and the maximum speed was just over 11 km day^−1^. While these migration speeds are relatively slow compared to the cruising speeds of other (typically surface-dwelling) ocean migrating fishes, they are consistent with other studies of anguillid eels^12,20,27,28^. The data therefore support the hypothesis that, rather than make a rapid migration to spawn at the earliest opportunity, European eels may instead make a long, slow spawning migration at depth that conserves their energy and reduces mortality risk^20^. This timing would enable the completion of their reproductive maturation^20^ before they arrive at the spawning area in time for the peak of the second spawning period after the onset of their migration (Fig. 1C).

There has been a long quest to understand the migratory behaviour of anguillid eels and when Johannes Schmidt (1923)^1^ first identified the presumed spawning area, he stated:

Years of research rich in excitement and suspense: disappointment alternating with encouraging discoveries and periods of rapid progress with others during which the solution of the problem seemed wrapped in deeper darkness than ever

Although our study did not yet yield definitive evidence of the mechanism(s) of navigational influence such as perhaps ocean currents, olfactory cues, temperature fronts, magnetic fields or seamounts^29–33^, ours is the first direct evidence of migrating adult European eels reaching the presumed breeding place in the Sargasso Sea. This is an encouraging discovery that completes the map of the spawning migration route that has emerged over the last 10 years^17–21^ and offers some light on how to develop future work. The dramatic (95%) decline in juvenile recruitment since the 1980s of this critically endangered species^34–37^, highlights the importance of further research into all aspects of the life-cycle, including adult migration navigation mechanisms and spawning locations, to inform conservation measures that will lead to a sustainable recovery of the European eel population^13^.

## Methods

### Pop-up archival satellite tags (PSATs)

All eels were equipped with an X tag from Microwave Telemetry (www.microwavetelemetry.com) which is 120 mm long, with a 185 mm long antenna. The maximum diameter of the float is 33 mm. Weight in air is 45 g and net buoyancy in seawater is approximately 0.025 ±  0.006 N, corresponding to a negative weight of 2.6 g. The tag measures and stores pressure, temperature and light data every 2 min. A subset of these data are transmitted when the tags pop up at either a predetermined date or if any of the fail-safe devices are triggered, for example if a critical pressure is exceeded. The temperature measurement range is − 4° to 40 °C with a resolution of 0.23 °C. The depth range is 0 to 1300 m with a resolution varying between 0.34 and 5.4 m depending on the gain value which is automatically selected according to the depth measured at midnight each day. The time of release was programmed at 6 months (n = 10), 7 months (n = 10) or 1 year (n = 6) after deployment. The constant pressure release feature that detaches the tag if the depth reading remains within 3 m for a period of 4 days was deactivated for the first 20 days after deployment to avoid premature release with limited movements in shallow water. After a tag pops up, surface position and a time series of depth and temperature are transmitted from the tag to low Earth orbiting ARGOS satellites (http://www.argos-system.org/) from where they can be downloaded. More details on how data from the tag are coded and transmitted can be found at https://www.microwavetelemetry.com/data. The surfacing positions of tags that drifted at the surface before activating their communication with the ARGOS satellite system were back-calculated by determining the direction and velocity of surface currents observed after the tag’s first ARGOS transmission over the same period of time as the surface drift period.

### Eel capture and tagging

Wild adult ‘silver’ eels were captured during their Autumn downstream migration using fyke nets set in the freshwater reaches of rivers on the Azorean Islands of San Miguel and Flores. There is no commercial eel fishery in the Azores and very little was known about the population status of European eel, hence capture sites were identified from surveys conducted in 2017, 2018 and 2019 across the archipelago. Environmental DNA testing (Nature Metrics) at the survey locations confirmed the species as *A. anguilla*. Three eels caught between November 14th and 22nd 2018 on San Miguel that were large enough to be tagged (865 to 990 mm; 1.4 to 2.1 kg) were transferred to a trout farm on the island and held in a raceway until tagging and release on December 9th 2018. In 2019, 23 large eels (820 to 1019 mm length, 1.3 to 2.2 kg) captured on Flores between October 16th and November 25th were transferred to the raceway of a trout farm on the same island and held for a maximum of 32 days. Tagging occurred on two occasions in 2019; 16 and 7 eels were tagged and released on November 6th and 27th, respectively, bringing the total number of eels tagged to 26 (Table 1).

All eels selected for tagging were assumed to be female based on their lengths being far greater than those typically achieved by maturing males^30,38^. Maturation state was quantified using Pankhurst’s ocular index (OI)^39^ and Fin Index^38^.

The PSAT attachment procedure was conducted under anaesthesia (2-phenoxyethanol, 2 ml l^−1^) using a three-point stainless steel wire attachment inserted dorsally under the skin^40^. There were no mortalities during tagging or recovery. After recovery, eels were transported to the release location in individual tanks (70 L) to prevent entanglement of the external tags. In 2018, the release occurred at a bay on the southern coast of San Miguel (37.43011 °N, 25. 28276 °W). In 2019, it was at the sheltered southern end of a bay on the west coast of Flores (39.45937 °N, 31.26213 °W).

### Determining the fate of eels

All PSAT tags rise to the surface once they become detached. The data preceding the final ascent were assessed to determine the fate of the eel. These were classified as either (1) programmed release date, (2) premature detachment, causes unknown, (3) premature detachment due to exceeding tag depth limit, (4) predation identified by changes in temperature readings or behaviours consistent with ingestion by a marine mammal or surface-orientated ectotherm^20^, or (5) no data received.

### Migration speed, distance and routes

Since the mechanism of eel migration is not known, we assumed that eels travelled at a constant speed along a constant bearing between the release position and pop-up position, and that the distance travelled was the loxodromic distance (rhumb line) between the points. Migration speed (km day^−1^) was calculated by dividing distance travelled by the number of days at liberty, excepting the data from eels that were predated (n = 2). To assess whether the remaining migration data (n = 21, from eels released in both 2018 and 2019) should be aggregated, the data on speed were split into three groups based on time at liberty (< 120 day, 120 to 240 day, and > 240 day) and differences in average migratory speed over the course of the migration were assessed using a Kruskal–Wallis test. The same approach was taken to assess differences in migratory bearing for eels released from Flores in 2019 (n = 19: eels released from San Miguel were excluded as the difference in release location would have a significant effect on the calculation of bearing). Although migratory speed reduced with time spent at liberty, this difference was not significant (H = 2.7, *p* > 0.05, *df* = 2). The migratory bearing did not differ significantly among groups (H = 0.17, *p* > 0.05, *df* = 2). Projected location at the time of presumed peak spawning (14th February, based on analysis of larval data reported in Righton et al. 2016^20^) was estimated using the average migration bearing of all eels released in 2019 and the migration speed of all eels.

### Larval data

Historical data on eel larvae, which were compiled by J. D. McCleave and hosted by the International Council for the Exploration of the Sea (ICES Eggs and larvae (ices.dk)) were analysed to determine the location of the smallest *A. anguilla* leptocephalus larvae in the Sargasso Sea. The database covers the results of eel larvae surveys from 1862 to 2007, comprising a total of 2375 hauls made using varying plankton and larval nets, and yielding a catch of more than 32,000 *A. anguilla* or *Anguilla rostrata* leptocephali. The stations were spread across the North Atlantic but concentrated in the presumed spawning area in the Sargasso Sea. The data were grouped into a 1° grid and each grid cell was classified according to the minimum size of the larvae found within it.

### Ethical approval

This animal study was reviewed and approved by the Zoological Society of London Ethics Committee. Field work was covered by the appropriate Azores Government licences, namely from the Directories of Environment (Licences 989/2017/DRA, 97/2918/DRA and 33/2019/DRA) and Science and Technology (CCPI 39/2917/DRCT, 45/2018/DRCT and 20/2019/DRCT). All methods were performed in accordance with the relevant guidelines and regulations. The study is reported in accordance with ARRIVE guidelines.

## Data Availability

The datasets generated during and/or analysed during the current study are available from the corresponding authors on reasonable request.
