# Author Correction: First direct evidence of adult European eels migrating to their breeding place in the Sargasso Sea

**DOI:** 10.1038/s41598-022-26117-x

**Published:** 2022-12-15

**Authors:** Rosalind M. Wright, Adam T. Piper, Kim Aarestrup, Jose M. N. Azevedo, George Cowan, Andy Don, Matthew Gollock, Sara Rodriguez Ramallo, Randolph Velterop, Alan Walker, Håkan Westerberg, David Righton

**Affiliations:** 1 Environment Agency, Threshelfords Business Park, Feering, Essex, CO5 9SE UK; 2 Institute of Zoology, Regents Park, London, NW1 4RY UK; 3 Technical University of Denmark, National Institute of Aquatic Resources, Vejlsoevej 39, 8600 Silkeborg, Denmark; 4 Centre for Ecology, Evolution and Environmental Changes and Faculdade de Ciências E Tecnologia, Universidade Dos Açores, Rua Mãe de Deus, 9500-321 Ponta Delgada, Azores Portugal; 5 Raufarhöfn, Iceland; 6 Environment Agency, Rivers House, East Quay, Bridgwater, TA6 4YS UK; 7 Zoological Society of London, Regents Park, London, NW1 4RY UK; 8 Natural England, Sterling House, Dix’s Field, Exeter, EX1 1QA UK; 9 The Centre for Environment, Fisheries and Aquaculture Science, Pakefield Road, Lowestoft, Suffolk, NR33 0HT UK; 10 Department of Aquatic Resources, Institute of Freshwater Research, Swedish University of Agricultural Sciences, Stångholmsvägen 2, 178 93 Drottningholm, Sweden

Correction to: *Scientific Reports* 10.1038/s41598-022-19248-8, published online 13 October 2022

The original version of this Article contained an error.

In the Results section,

“Average migration speed ranged between 2.9 and 11.9 m day^−1^ (mean 6.8 km day^−1^ ± 2.2 s.d.).”

now reads:

“Average migration speed ranged between 2.9 and 11.9 km day^−1^ (mean 6.8 km day^−1^ ± 2.2 s.d.).”

The original Article has been corrected.

